# Displacing, squeezing, and time evolution of quantum states for nanoelectronic circuits

**DOI:** 10.1186/1556-276X-8-30

**Published:** 2013-01-15

**Authors:** Jeong Ryeol Choi, Byeong Jae Choi, Hyun Deok Kim

**Affiliations:** 1 Department of Radiologic TechnologyDaegu Health College, , Buk-gu, Daegu, 702-722, Republic of Korea; 2 School of Electronics and Electrical EngineeringDaegu University, , Gyeongsan, Gyeongbuk, 712-714, Republic of Korea; 3 School of Electronics Engineering, College of IT Engineering, Kyungpook National University, Daegu, 702-701, Republic of Korea

**Keywords:** Displaced squeezed number states, Electronic circuits, Nanoscale physics, Unitary transformation, Fluctuations of charges and currents

## Abstract

The time behavior of DSN (displaced squeezed number state) for a two-dimensional electronic circuit composed of nanoscale elements is investigated using unitary transformation approach. The original Hamiltonian of the system is somewhat complicated. However, through unitary transformation, the Hamiltonian became very simple enough that we can easily treat it. By executing inverse transformation for the wave function obtained in the transformed system, we derived the exact wave function associated to the DSN in the original system. The time evolution of the DSN is described in detail, and its corresponding probability density is illustrated. We confirmed that the probability density oscillates with time like that of a classical state. There are two factors that drive the probability density to oscillate: One is the initial amplitude of complementary functions, and the other is the external power source. The oscillation associated with the initial amplitude gradually disappears with time due to the dissipation raised by resistances of the system. These analyses exactly coincide with those obtained from classical state. The characteristics of quantum fluctuations and uncertainty relations for charges and currents are also addressed.

## Background

The technical range of nanoscale is 1 to 999 nm, but people often refer to nanosize when an element is smaller than about 100 nm, where quantum effects are dominant instead of classical ones. Nanophysics and nanoelectronics have been rapidly developed thanks to the advancement of relevant technologies such as crystal growth and lithography, which facilitate sophisticated experiments for nanosystems [1,2]. A recent conspicuous trend in the community of electronic device is that the integrated circuits and components are miniaturized towards atomic-scale dimensions [2]. We can confirm from many experiments and theories associated with nanoscale elements that the quantum effects become prominent when the transport dimension reaches a critical value which is the Fermi wavelength, while at the same situation, the classical theory for the motion of charges and currents is invalid. Not only quantum dot and quantum wire but also the quantum characteristics of electronic circuits involving nanoscale elements are important as a supporting theory for nanometer electronic technology and quantum information technology. For this reason, quantum effects in electronic circuits with nanoscale elements have been widely studied in recent years.

The simple quantum model of a lossless inductor-capacitor (LC) circuit have been suggested firstly by Louisell [3]. Zhang et al. investigated the quantum properties of two-dimensional electronic circuits which have no power source [4]. The quantum behavior of charges and currents for an LC circuit [5] and a resistor-inductor-capacitor (RLC) linear circuit [3] driven by a power source have been studied by several researchers. If a circuit contains resistance, the electronic energy of the system dissipates with time. In this case, the system is described by a time-dependent Hamiltonian. Another example of the systems described by time-dependent Hamiltonian is electronic circuits driven by time-varying power sources. The quantum problem of time-dependent Hamiltonian systems attracted great concern in the community of theoretical physics and chemistry for several decades [4,6,7].

The study of electronic characteristics of charge carriers in nanoelectronic circuits is basically pertained to a physical problem. There are plentiful reports associated with the physical properties of miniaturized two-loop (or two-dimensional) circuits [8-12] and more high multi-loop circuits [13-16] including their diverse variants. Various applications which use two-loop circuits include a switch-level resistor-capacitor (RC) model of an n-transistor (see Figure 3 of [8]), a design of a prototype of current-mode leapfrog ladder filters (Sect. 3 of [9]), and a port-Hamiltonian system [10], whereas higher loop circuits can be used as a transmission line model for multiwall carbon nanotube [13] and a filter circuit for electronic signals (Sect. 5 of [15]).

In this paper, we derive quantum solutions of a two-dimensional circuit coupled via RL and investigate its displaced squeezed number state (DSN) [17]. We suppose that the system is composed of nanoscale elements and driven by a time-varying power source. The unitary transformation method which is very useful when treating time-dependent Hamiltonian systems in cases like this will be used. We can obtain the wave functions of DSN by first applying the squeezing operator in those of the number state and then applying the unitary displacement operator. Under displaced quantum states of circuit electrodynamics, conducting charges (or currents) exhibit collective classical-like oscillation. The fluctuations and uncertainty relations for charges and currents will be evaluated in the DSN without approximation.

Displaced squeezed number states, which are the main topic in this work, belong to nonclassical states that have been objects of many investigations. The statistical properties of these states exhibit several pure quantum effects which have no classical analogues, including the interference in the phase space [18], the revival/collapse phenomenon [19], and sub-Poissonian statistics [20]. The position representation of these states with overall phases is derived by Moller et al. for the simple harmonic oscillator by employing geometric operations in phase space [17]. The effects of quantum interference between two distinct DSNs prepared to be out of phase with respect to each other are investigated by Faisal et. al., discussing various nonclassical properties in connection with quantum number distribution, purity, quadrature squeezing, W-function, etc. [21].

## Methods and results

### Simplification via unitary transformation

**Figure 1:**
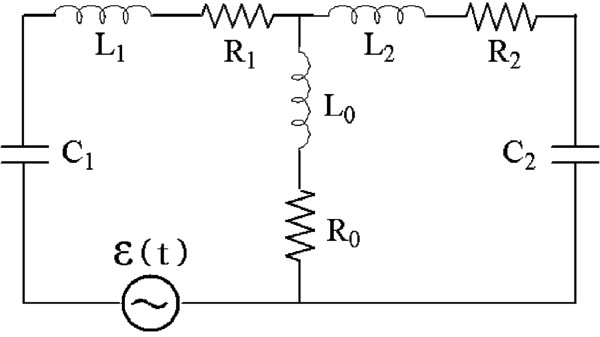
**Electronic circuit.** This is the diagram of a two-dimensional electronic circuit composed of nanoscale elements.

Let us consider two loops of RLC circuit, whose elements are nanosized, that are coupled with each other via inductance and resistance as shown in Figure 1. Using Kirchhoff’s law, we obtain the classical equations of motion for charges of the system [4]:

(1) $$\begin{array}{ll} L_{1}\frac{d^{2}q_{1}}{dt^{2}} & +R_{1}\frac{dq_{1}}{\text{dt}}+\frac{q_{1}}{C_{1}}+L_{0}\left(\frac{d^{2}q_{1}}{dt^{2}}-\frac{d^{2}q_{2}}{dt^{2}}\right)\\ & +R_{0}\left(\frac{dq_{1}}{\text{dt}}-\frac{dq_{2}}{\text{dt}}\right)=E(t), \end{array}$$

(2) $$\begin{array}{ll} L_{2}\frac{d^{2}q_{2}}{dt^{2}} & +R_{2}\frac{dq_{2}}{\text{dt}}+\frac{q_{2}}{C_{2}}-L_{0}\left(\frac{d^{2}q_{1}}{dt^{2}}-\frac{d^{2}q_{2}}{dt^{2}}\right)\\ & -R_{0}\left(\frac{dq_{1}}{\text{dt}}-\frac{dq_{2}}{\text{dt}}\right)=0, \end{array}$$

where *q*_*j*_ (*j*=1,2; hereafter, this convention will be used for all *j*) are charges stored in the capacitances *C*_*j*_, respectively, and E(t) is an arbitrary time-varying voltage source connected in loop 1. If we consider not only the existence of E(t) but also the mixed appearance of *q*_1_ and *q*_2_ in these two equations, it may be not an easy task to treat the system directly. If the scale of resistances are sufficiently large, the system is described by an overdamped harmonic oscillator, whereas the system becomes an underdamped harmonic oscillator in the case of small resistances. In this paper, we consider only the underdamped case.

For convenience, we suppose that *R*_0_/*L*_0_=*R*_1_/*L*_1_=*R*_2_/*L*_2_≡*β*. Then, the classical Hamiltonian of the system can be written as

(3) $$\begin{array}{ll} H\;\;=\;\; & e^{-\mathrm{\beta t}}\left(\frac{p_{1}^{2}}{2L_{1}}+\frac{p_{2}^{2}}{2L_{2}}-\frac{1}{2}{\left(k_{1}p_{1}-k_{2}p_{2}\right)}^{2}\right)\\ & +e^{\mathrm{\beta t}}\left(\frac{q_{1}^{2}}{2C_{1}}+\frac{q_{2}^{2}}{2C_{2}}-E(t)q_{1}\right), \end{array}$$

where *p*_*j*_ are canonical currents of the system, and *k*_*j*_=(1/*L*_*j*_)(1/*L*_0_+1/*L*_1_+1/*L*_2_)^−1/2^. From Hamilton’s equations, we can easily see that *p*_*j*_ are given by

(4) $$p_{1}=e^{\mathrm{\beta t}}[(L_{0}+L_{1}){\dot{q}}_{1}-L_{0}{\dot{q}}_{2}],$$

(5) $$p_{2}=e^{\mathrm{\beta t}}[(L_{0}+L_{2}){\dot{q}}_{2}-L_{0}{\dot{q}}_{1}].$$

If we replace classical variables *q*_*j*_ and *p*_*j*_ in Equation 3 with their corresponding operators, ${\hat{q}}_{j}$ and ${\hat{p}}_{j}$, the classical Hamiltonian becomes quantum Hamiltonian:

(6) $$\begin{array}{ll} Ĥ\;\;= & \;\;e^{-\mathrm{\beta t}}\left(\frac{{\hat{p}}_{1}^{2}}{2L_{1}}+\frac{{\hat{p}}_{2}^{2}}{2L_{2}}-\frac{1}{2}{\left(k_{1}{\hat{p}}_{1}-k_{2}{\hat{p}}_{2}\right)}^{2}\right)\\ & +e^{\mathrm{\beta t}}\left(\frac{{\hat{q}}_{1}^{2}}{2C_{1}}+\frac{{\hat{q}}_{2}^{2}}{2C_{2}}-E(t){\hat{q}}_{1}\right), \end{array}$$

where ${\hat{p}}_{j}=-\mathrm{i\hbar \partial}/\partial q_{j}$. Now, we are going to transform $\hat{H}$ into a simple form using the unitary transformation method, developed in [6] for a two-loop LC circuit, in order to simplify the problem. Let us first introduce a unitary operator

(7) $${Û}_{A}={Û}_{A1}\;{Û}_{A2},$$

where

(8) $$\begin{array}{ll} {Û}_{A1}= & \exp\left\{\left[\frac{i}{\hbar }\ln{\left(\frac{C_{2}}{C_{1}}\right)}^{1/8}+i\frac{\beta }{4\hbar }t\right]({\hat{p}}_{1}{\hat{q}}_{1}+{\hat{q}}_{1}{\hat{p}}_{1})\right\}\\ & \times \exp\left\{\left[\frac{i}{\hbar }\ln{\left(\frac{C_{1}}{C_{2}}\right)}^{1/8}+i\frac{\beta }{4\hbar }t\right]({\hat{p}}_{2}{\hat{q}}_{2}+{\hat{q}}_{2}{\hat{p}}_{2})\right\}, \end{array}$$

(9) $$\begin{array}{l} {Û}_{A2}=\exp\left[-\frac{\mathrm{i\varphi}}{\hbar }({\hat{p}}_{1}{\hat{q}}_{2}-{\hat{p}}_{2}{\hat{q}}_{1})\right], \end{array}$$

with

(10) $$\begin{array}{l} \varphi \;=\;\frac{1}{2}\overset{-1}{\tan}\;\;\left\{2k_{1}k_{2}\;{\left[\sqrt{\frac{C_{2}}{C_{1}}}\left(k_{1}^{2}\;-\;\frac{1}{L_{1}}\right)\;-\;\sqrt{\frac{C_{1}}{C_{2}}}\left(k_{2}^{2}\;-\;\frac{1}{L_{2}}\right)\;\right]}^{-1}\right\}. \end{array}$$

Using Equation 7, we can transform the Hamiltonian such that

(11) $${Ĥ}_{A}={Û}_{A}^{-1}Ĥ{Û}_{A}-\mathrm{i\hbar}{Û}_{A}^{-1}\frac{\partial {Û}_{A}}{\mathrm{\partial t}}.$$

A straightforward algebra after inserting Equation 6 into the above equation gives

(12) $${Ĥ}_{A}({\hat{q}}_{1},{\hat{p}}_{1},{\hat{q}}_{2},{\hat{p}}_{2},t)={Ĥ}_{A1}({\hat{q}}_{1},{\hat{p}}_{1},t)+{Ĥ}_{A2}({\hat{q}}_{2},{\hat{p}}_{2},t),$$

where

(13) $$\begin{array}{ll} {Ĥ}_{\text{Aj}}({\hat{q}}_{j},{\hat{p}}_{j},t)\;\;=\;\; & \frac{{\hat{p}}_{j}^{2}}{2\mu_{j}}+\frac{\beta }{4}({\hat{q}}_{j}{\hat{p}}_{j}+{\hat{p}}_{j}{\hat{q}}_{j})+\frac{1}{2\sqrt{C_{1}C_{2}}}{\hat{q}}_{j}^{2}\\ & -{\hat{q}}_{j}E(t){\left(\frac{C_{1}}{C_{2}}\right)}^{1/4}e^{\mathrm{\beta t}/2}\cos\left(\varphi -\frac{\Pi }{2}\delta_{j,2}\right), \end{array}$$

with

(14) $$\begin{array}{ll} \mu_{1}\;\;= & \;\;\left[\sqrt{\frac{C_{2}}{C_{1}}}\left(\frac{1}{L_{1}}-k_{1}^{2}\right)\overset{2}{\cos}\varphi +\sqrt{\frac{C_{1}}{C_{2}}}\left(\frac{1}{L_{2}}-k_{2}^{2}\right)\overset{2}{\sin}\varphi \right)\\ & {\left(-k_{1}k_{2}\sin(2\varphi )\right]}^{-1}, \end{array}$$

(15) $$\begin{array}{ll} \mu_{2}\;\;=\;\; & \left[\sqrt{\frac{C_{2}}{C_{1}}}\left(\frac{1}{L_{1}}-k_{1}^{2}\right)\overset{2}{\sin}\varphi +\sqrt{\frac{C_{1}}{C_{2}}}\left(\frac{1}{L_{2}}-k_{2}^{2}\right)\overset{2}{\cos}\varphi \right)\\ & {\left(+k_{1}k_{2}\sin(2\varphi )\right]}^{-1}. \end{array}$$

One can see from Equation 13 that the coupled term involving ${\hat{q}}_{1}{\hat{q}}_{2}$ in the original Hamiltonian is decoupled through this transformation. However, the Hamiltonian still contains linear terms that are expressed in terms of ${\hat{q}}_{j}E(t)$, which are hard to handle when developing a quantum theory of the system. To remove these terms, we introduce another unitary operator of the form

(16) $${Û}_{B}={Û}_{B1}\;{Û}_{B2},$$

(17) $$\begin{array}{ll} {Û}_{B1}\;\;=\;\; & \exp\left[\frac{i}{\hbar }[p_{1p}(t)\;{\hat{q}}_{1}+p_{2p}(t)\;{\hat{q}}_{2}]\right]\\ & \times \exp\left[-\frac{i}{\hbar }[q_{1p}(t)\;{\hat{p}}_{1}+q_{2p}(t)\;{\hat{p}}_{2}]\right], \end{array}$$

(18) $${Û}_{B2}=\exp\left[-\frac{\mathrm{i\beta}}{4\hbar }(\mu_{1}{\hat{q}}_{1}^{2}+\mu_{2}{\hat{q}}_{2}^{2})\right],$$

where *q*_*jp*_(*t*) and *p*_*jp*_(*t*) are classical particular solutions of the firstly transformed system described by ${Ĥ}_{A}$ in the charge and the current spaces, respectively. From basic Hamiltonian dynamics with the use of Equation 12, we see that *q*_*jp*_(*t*) and *p*_*jp*_(*t*) satisfy the time-dependent classical equations that are given by

(19) $${\ddot{q}}_{\text{jp}}(t)+\omega_{j}^{2}q_{\text{jp}}(t)-\frac{E(t)}{\mu_{j}}\sqrt[{4}]{\frac{C_{1}}{C_{2}}}e^{\mathrm{\beta t}/2}\cos\left(\varphi -\frac{\Pi }{2}\delta_{j,2}\right)=0,$$

(20) $${\ddot{p}}_{\text{jp}}(t)+\omega_{j}^{2}p_{\text{jp}}(t)-\dot{E}(t)\sqrt[{4}]{\frac{C_{1}}{C_{2}}}e^{\mathrm{\beta t}/2}\cos\left(\varphi -\frac{\Pi }{2}\delta_{j,2}\right)=0,$$

where

(21) $$\omega_{j}={\left(\frac{1}{\mu_{j}\sqrt{C_{1}C_{2}}}-\frac{\beta^{2}}{4}\right)}^{1/2}.$$

Then, the second transformation yields

(22) $$\begin{array}{ll} {Ĥ}_{B}({\hat{q}}_{1},{\hat{p}}_{1},{\hat{q}}_{2},{\hat{p}}_{2},t) & ={Û}_{B}^{-1}{Ĥ}_{A}{Û}_{B}-\mathrm{i\hbar}{Û}_{B}^{-1}\frac{\partial {Û}_{B}}{\mathrm{\partial t}}\\ & ={Ĥ}_{B1}({\hat{q}}_{1},{\hat{p}}_{1},t)+{Ĥ}_{B2}({\hat{q}}_{2},{\hat{p}}_{2},t), \end{array}$$

where

(23) $${Ĥ}_{\text{Bj}}({\hat{q}}_{j},{\hat{p}}_{j},t)=\frac{{\hat{p}}_{j}^{2}}{2\mu_{j}}+\frac{1}{2}\mu_{j}\omega_{j}^{2}{\hat{q}}_{j}^{2}+L_{\text{jp}}(t),$$

with

(24) $$L_{\text{jp}}(t)=\frac{1}{2}\mu_{j}{\dot{q}}_{\text{jp}}^{2}(t)-\frac{1}{2\sqrt{C_{1}C_{2}}}q_{\text{jp}}^{2}(t).$$

The finally transformed Hamiltonian, Equation 22, is very simple and no longer involves linear terms that contain E(t). If we neglect $L_{\text{jp}}(t)$, this is exactly the same as that of the two-dimensional simple harmonic oscillator of frequencies *ω*_*j*_. We will use this formula in order to develop DSN, which is a typical nonclassical quantum state.

If we regard that the transformed Hamiltonian is very simple, the quantum dynamics in the transformed system may be easily developed. Let us write the Schrödinger equations for elements of the transformed Hamiltonian as

(25) $$\mathrm{i\hbar}\frac{\partial \psi_{n_{j}}^{B}(q_{j},t)}{\mathrm{\partial t}}={Ĥ}_{\text{Bj}}\psi_{n_{j}}^{B}(q_{j},t),$$

where $\psi_{n_{j}}^{B}(q_{j},t)$ represent number state wave functions for each component of the decoupled systems described by ${Ĥ}_{\text{Bj}}$.

By means of the usual annihilation operator,

(26) $${â}_{j}=\sqrt{\frac{\mu_{j}\omega_{j}}{2\hbar }}{\hat{q}}_{j}+\frac{i{\hat{p}}_{j}}{\sqrt{2\hbar \mu_{j}\omega_{j}}},$$

and the creation operator ${â}_{j}^{‡}$ defined as the Hermitian adjoint of ${â}_{j}$, one can identify the initial wave functions of the transformed system in number state such that

(27) $$\psi_{n_{1},n_{2}}^{B}(q_{1},q_{2},0)=\psi_{n_{1}}^{B}(q_{1},0)\psi_{n_{2}}^{B}(q_{2},0),$$

where

(28) $$\begin{array}{ll} \psi_{n_{j}}^{B}(q_{j},0)\;\;=\;\; & {\left(\frac{\mu_{j}\omega_{j}}{\hbar \Pi }\right)}^{1/4}\frac{1}{\sqrt{2^{n_{j}}n_{j}!}}H_{n_{j}}\left[{\left(\frac{\mu_{j}\omega_{j}}{\hbar }\right)}^{1/2}q_{j}\right]\\ & \times \exp\left(-\frac{\mu_{j}\omega_{j}}{2\hbar }\overset{2}{\underset{j}{q}}\right). \end{array}$$

This formula of wave functions will be used in the next section in order to derive the DSN of the system.

### Displaced squeezed number state

The DSNs are defined by first squeezing the number states and then displacing them. Like squeezed states, DSNs exhibit nonclassical properties of the quantum field in which the fluctuation of a certain observable can be less than that in the vacuum state. This state is a generalized quantum state for dynamical systems and, in fact, equivalent to excited two-photon coherent states in quantum optics. If we consider that DSNs generalize and combine the features of well-known important states such as displaced number states (DNs) [22], squeezed number states [23], and two-photon coherent states (non-excited) [24], the study of DSNs may be very interesting. Different aspects of these states, including quantal statistics, entropy, entanglement, and position space representation with the correct overall phase, have been investigated in [17,23,25].

To obtain the DSN in the original system, we first derive the DSN in the transformed system according to its exact definition. Then, we will transform it inversely into that of the original system. The squeeze operator in the transformed system is given by

(29) $${Ŝ}_{j}(z_{j})=\exp\left[-\frac{1}{2}(\overset{*}{\underset{j}{z}}{â}_{j}^{2}-z_{j}{â}_{j}^{‡2})\right],$$

where

(30) $$z_{j}=r_{j}e^{i\phi_{j}}.$$

Using the Baker-Campbell-Hausdorff relation that is given by [26]

(31) $$\begin{array}{ll} \exp & \left\{\frac{1}{2\hbar }[a{\hat{q}}^{2}+\text{ic}(\hat{q}\hat{p}+\hat{p}\hat{q})-b{\hat{p}}^{2}]\right\}=\frac{1}{\sqrt{\cosh\theta -\frac{c}{\theta }\sinh\theta }}\\ & \;\times \exp\left[\frac{a}{2\theta \hbar }\sinh\theta {\left(\cosh\theta -\frac{c}{\theta }\sinh\theta \right)}^{-1}{\hat{q}}^{2}\right]\\ & \;\times \exp\left[-\frac{i}{\hbar }\ln\left(\cosh\theta -\frac{c}{\theta }\sinh\theta \right)\hat{q}\hat{p}\right]\\ & \;\times \exp\left[-\frac{b}{2\theta \hbar }\sinh\theta {\left(\cosh\theta -\frac{c}{\theta }\sinh\theta \right)}^{-1}{\hat{p}}^{2}\right], \end{array}$$

where $\theta =\sqrt{c^{2}-\text{ab}}$, the squeeze operator can be rewritten as

(32) $$\begin{array}{ll} {Ŝ}_{j}(z_{j})\;\;=\;\; & \frac{1}{\sqrt{\cosh r_{j}+\cos\phi_{j}\sinh r_{j}}}\\ & \times \exp\left[\frac{i\mu_{j}\omega_{j}}{2\hbar }\frac{\sin\phi_{j}\sinh r_{j}}{\cosh r_{j}+\cos\phi_{j}\sinh r_{j}}\overset{2}{\underset{j}{\hat{q}}}\right]\\ & \times \exp\left[-\frac{i}{\hbar }\ln\left(\cosh r_{j}+\cos\phi_{j}\sinh r_{j}\right){\hat{q}}_{j}{\hat{p}}_{j}\right]\\ & \times \exp\left[-\frac{i}{2\mu_{j}\omega_{j}\hbar }\frac{\sin\phi_{j}\sinh r_{j}}{\cosh r_{j}+\cos\phi_{j}\sinh r_{j}}\overset{2}{\underset{j}{\hat{p}}}\right]. \end{array}$$

Let us express the DSN in the transformed system in the form

(33) $$\psi_{s,n_{1},n_{2}}^{B}(q_{1},q_{2},t)=\psi_{s,n_{1}}^{B}(q_{1},t)\psi_{s,n_{2}}^{B}(q_{2},t),$$

where $\psi_{s,n_{j}}^{B}(q_{j},t)$ represent two decoupled states which are drivable from

(34) $$\psi_{s,n_{j}}^{B}(q_{j},t)={\hat{T}}_{\text{Bj}}({\hat{q}}_{j},{\hat{p}}_{j},t){\hat{D}}_{j}(\alpha_{j}){Ŝ}_{j}(z_{j})\psi_{n_{j}}^{B}(q_{j},0).$$

Here, ${\hat{D}}_{j}(\alpha_{j})$ are displacement operators in the transformed system, which are given by

(35) $${\hat{D}}_{j}(\alpha_{j})=\exp(\alpha_{j}{â}_{j}^{‡}-\alpha_{j}^{*}{â}_{j}),$$

where *α*_*j*_ is an eigenvalue of ${â}_{j}$ at initial time. By considering Equation 26, we can confirm that

(36) $$\alpha_{j}=\sqrt{\frac{\mu_{j}\omega_{j}}{2\hbar }}q_{\text{jc}}(0)+\frac{ip_{\text{jc}}(0)}{\sqrt{2\hbar \mu_{j}\omega_{j}}},$$

where *q*_*jc*_(*t*) and *p*_*jc*_(*t*) are classical solutions of the equation of motion in charge and current spaces, respectively, for the finally transformed system. If we regard that the complementary functions [27] of the equation of motion in the firstly transformed system are the same as the classical solutions of the finally transformed system, *q*_*jc*_(*t*) and *p*_*jc*_(*t*) can also be complementary functions of the firstly transformed system. The other operators ${\hat{T}}_{\text{Bj}}({\hat{q}}_{j},{\hat{p}}_{j},t)$ are time-displacement operators:

(37) $${\hat{T}}_{\text{Bj}}({\hat{q}}_{j},{\hat{p}}_{j},t)=\exp\left(-\frac{i}{\hbar }\int_{0}^{t}{Ĥ}_{\text{Bj}}({\hat{q}}_{j},{\hat{p}}_{j},t^{\prime })dt^{\prime }\right).$$

At first, the action of squeezing operator in wave functions of the initial number state gives

(38) $$\begin{array}{ll} {Ŝ}_{j}(z_{j})\psi_{n_{j}}^{B}(q_{j},0)= & {\left(\frac{\mu_{j}\omega_{j}}{\hbar \Pi }\right)}^{1/4}\;\;\frac{1}{\sqrt{2^{n_{j}}n_{j}!}}\sqrt{\frac{G_{\text{bj}}^{n_{j}}}{G_{\text{aj}}}}H_{n_{j}}\left[{\left(\frac{\mu_{j}\omega_{j}}{\hbar G_{\text{cj}}}\right)}^{1/2}q_{j}\right]\\ & \times \exp\left(-\frac{\mu_{j}\omega_{j}}{2\hbar }G_{\text{dj}}q_{j}^{2}\right), \end{array}$$

where

(39) $$\;G_{\text{aj}}=\cosh r_{j}+e^{i\phi_{j}}\sinh r_{j},$$

(40) $$\;G_{\text{bj}}=\frac{\cosh r_{j}+e^{-i\phi_{j}}\sinh r_{j}}{\cosh r_{j}+e^{i\phi_{j}}\sinh r_{j}},$$

(41) $$\;G_{\text{cj}}=\overset{2}{\cosh}r_{j}+\overset{2}{\sinh}r_{j}+2\cos\phi_{j}\cosh r_{j}\sinh r_{j},$$

(42) $$\;G_{\text{dj}}=\frac{1-i\sin\phi_{j}\sinh r_{j}(\cosh r_{j}+e^{i\phi_{j}}\sinh r_{j})}{\left(\cosh r_{j}+\cos\phi_{j}\sinh r_{j})(\cosh r_{j}+e^{i\phi_{j}}\sinh r_{j}\right)}.$$

The evaluation of the other actions of the operators in Equation 34 may be easily performed using Equation 31 and the relation [28]

(43) $$\;\begin{array}{l} \exp\left[c\hbar^{2}\frac{\partial^{2}}{\partial q^{2}}\right]h(q)=\frac{1}{\sqrt{4\Pi \hbar^{2}c}}\int_{-\infty }^{\infty }\exp\left[-\frac{{\left(y-q\right)}^{2}}{4c\hbar^{2}}\right]h(y)\text{dy}, \end{array}$$

together with the eighth formula of 7.374 in [29] (see Appendix Appendix 1), yielding

(44) $$\begin{array}{ll} \psi_{s,n_{j}}^{B}(q_{j},t)\;\;=\;\; & \sqrt[{4}]{\frac{\mu_{j}\omega_{j}}{\hbar \Pi }}\frac{1}{\sqrt{2^{n_{j}}n_{j}!}}\sqrt{\frac{{\left(h_{\text{bj}}G_{\text{bj}}\right)}^{n_{j}}}{h_{\text{aj}}G_{\text{aj}}}}\\ & \times H_{n_{j}}\left[\sqrt{\frac{\mu_{j}\omega_{j}}{\hbar h_{\text{aj}}^{2}h_{\text{bj}}G_{\text{cj}}}}[q_{j}-q_{\text{jc}}(t)]\right]\\ & \times \exp\left\{-\frac{\mu_{j}\omega_{j}}{2\hbar h_{\text{aj}}}\left[[G_{\text{dj}}\cos(\omega_{j}t)+i\sin(\omega_{j}t)]q_{j}^{2}\right)\right)\\ & \;\;\;-2q_{j}\left(G_{\text{dj}}q_{\text{jc}}(0)+i\frac{p_{\text{jc}}(0)}{\omega_{j}\mu_{j}}\right)\\ & \;\;\;\left(\left(+q_{\text{jc}}^{2}(0)G_{\text{dj}}\cos(\omega_{j}t)\right]\right\}\\ & \times \exp\left[-\frac{i\overset{2}{\underset{\text{jc}}{p}}(0)\sin(\omega_{j}t)}{2\mu_{j}\omega_{j}h_{\text{aj}}\hbar }-i\frac{q_{\text{jc}}(0)p_{\text{jc}}(0)}{\hbar }\right)\\ & \;\;\;\left(\times \left(\frac{1}{2}-i\frac{G_{\text{dj}}\sin(\omega_{j}t)}{h_{\text{aj}}}\right)\right]\\ & \times \exp\left[-\frac{i}{\hbar }\overset{t}{\underset{0}{\int }}L_{\text{jp}}(t^{\prime })dt^{\prime }\right], \end{array}$$

where

(45) $$h_{\text{aj}}=\cos(\omega_{j}t)+iG_{\text{dj}}\sin(\omega_{j}t),$$

(46) $$h_{\text{bj}}=1-\frac{2i\sin(\omega_{j}t)}{h_{\text{aj}}G_{\text{cj}}}.$$

Here, the time evolution of complementary functions are

(47) $$q_{\text{jc}}(t)=q_{\text{jc}}(0)\cos(\omega_{j}t)+\frac{p_{\text{jc}}(0)}{\mu_{j}\omega_{j}}\sin(\omega_{j}t),$$

(48) $$p_{\text{jc}}(t)=p_{\text{jc}}(0)\cos(\omega_{j}t)-\mu_{j}\omega_{j}q_{\text{jc}}(0)\sin(\omega_{j}t).$$

The transformed system reduces to a two-dimensional undriven simple harmonic oscillator in the limit E(t)=0. Our result in Equation 44 is exact, and in this limit, we can easily confirm that some errors in Equation 45 in [30] are corrected (see Appendix Appendix 2).

The wave function associated to the DSN in the transformed system will be transformed inversely to that of the original system in order to facilitate full study in the original system. This is our basic strategy. Thus, we evaluate the DSN in the original system from

(49) $$\psi_{s,n,m}(q_{1},q_{2},t)={Û}_{A}{Û}_{B}\psi_{s,n,m}^{B}(q_{1},q_{2},t).$$

Using the unitary operators given in Equations 7 and 16, we derive

(50) $$\begin{array}{rcll} \psi_{s,n_{1},n_{2}}(q_{1},q_{2},t) & & =\sqrt[{4}]{\frac{\mu_{1}\mu_{2}\omega_{1}\omega_{2}}{\hbar^{2}\Pi^{2}}}\frac{1}{\sqrt{2^{n_{1}+n_{2}}n_{1}!n_{2}!}}\sqrt{\frac{{\left(h_{b1}G_{b1}\right)}^{n_{1}}{\left(h_{b2}G_{b2}\right)}^{n_{2}}}{h_{a1}G_{a1}h_{a2}G_{a2}}}e^{\mathrm{\beta t}/2} & \\ & & \times \exp\left[\frac{i}{\hbar }e^{\mathrm{\beta t}/2}[p_{1p}(t)\;Q_{1}+p_{2p}(t)\;Q_{2}]\right]H_{n_{1}}\left[\sqrt{\frac{\mu_{1}\omega_{1}}{\hbar h_{a1}^{2}h_{b1}G_{c1}}}[e^{\mathrm{\beta t}/2}Q_{1}-q_{1p}(t)-q_{1c}(t)]\right] & \\ & & \times H_{n_{2}}\left[\sqrt{\frac{\mu_{2}\omega_{2}}{\hbar h_{a2}^{2}h_{b2}G_{c2}}}[e^{\mathrm{\beta t}/2}Q_{2}-q_{2p}(t)-q_{2c}(t)]\right] & \\ & & \times \exp\left\{-\overset{2}{\underset{j=1}{\sum }}\frac{\mu_{j}}{2\hbar }\left[\left(\frac{\omega_{j}}{h_{\text{aj}}}[G_{\text{dj}}\cos(\omega_{j}t)+i\sin(\omega_{j}t)]+\frac{\mathrm{i\beta}}{2}\right){\left[e^{\mathrm{\beta t}/2}Q_{j}-q_{\text{jp}}(t)\right]}^{2}\right]\right\} & \\ & & \times \exp\left\{\overset{2}{\underset{j=1}{\sum }}\frac{\mu_{j}\omega_{j}}{2\hbar h_{\text{aj}}}\left[2[e^{\mathrm{\beta t}/2}Q_{j}-q_{\text{jp}}(t)]\left(G_{\text{dj}}q_{\text{jc}}(0)+i\frac{p_{\text{jc}}(0)}{\omega_{j}\mu_{j}}\right)-\overset{2}{\underset{\text{jc}}{q}}(0)G_{\text{dj}}\cos(\omega_{j}t)\right]\right\} & \\ & & \times \exp\left\{-\overset{2}{\underset{j=1}{\sum }}\left[\frac{i\overset{2}{\underset{\text{jc}}{p}}(0)\sin(\omega_{j}t)}{2\mu_{j}\omega_{j}h_{\text{aj}}\hbar }+i\frac{q_{\text{jc}}(0)p_{\text{jc}}(0)}{\hbar }\left(\frac{1}{2}-i\frac{G_{\text{dj}}\sin(\omega_{j}t)}{h_{\text{aj}}}\right)\right]\right\} & \\ & & \times \exp\left[-\frac{i}{\hbar }\int_{0}^{t}[L_{1p}(t^{\prime })+L_{2p}(t^{\prime })]dt^{\prime }\right]. & \end{array}$$

This is the full expression of the time evolution of wave functions for the DSN. If we let *r*→0, the squeezing effects disappear, and consequently, the system becomes DN. Of course the above equation reduces, in this limit, to that of the DN.

To see the time behavior of this state, we take a sinusoidal signal as a power source, which is represented as

(51) $$E(t)=E_{0}\cos(\mathrm{\Omega t}+\delta ).$$

Then, the solution of Equations 19 and 20 is given by

(52) $$\begin{array}{ll} q_{1p}(t)=\frac{M_{1}(t)}{\mu_{1}}\cos\varphi & \left[\left(\beta^{2}-4\Omega^{2}+4\omega_{1}^{2})\cos(\mathrm{\Omega t}+\delta \right)\right)\\ & \left(+4\beta \Omega \sin(\mathrm{\Omega t}+\delta )\right], \end{array}$$

(53) $$\begin{array}{ll} p_{1p}(t)=-M_{1}(t)\Omega \cos\varphi & \left[\left(\beta^{2}-4\Omega^{2}+4\omega_{1}^{2})\sin(\mathrm{\Omega t}+\delta \right)\right)\\ & \left(-4\beta \Omega \cos(\mathrm{\Omega t}+\delta )\right], \end{array}$$

(54) $$\begin{array}{ll} q_{2p}(t)=\frac{M_{2}(t)}{\mu_{2}}\sin\varphi & \left[\left(\beta^{2}-4\Omega^{2}+4\omega_{2}^{2})\cos(\mathrm{\Omega t}+\delta \right)\right)\\ & \left(+4\beta \Omega \sin(\mathrm{\Omega t}+\delta )\right], \end{array}$$

(55) $$\begin{array}{ll} p_{2p}(t)=-M_{2}(t)\Omega \sin\varphi & \left[\left(\beta^{2}-4\Omega^{2}+4\omega_{2}^{2})\sin(\mathrm{\Omega t}+\delta \right)\right)\\ & \left(-4\beta \Omega \cos(\mathrm{\Omega t}+\delta )\right], \end{array}$$

where

(56) $$M_{j}(t)=\sqrt[{4}]{\frac{C_{1}}{C_{2}}}\frac{4E_{0}e^{\mathrm{\beta t}/2}}{\beta^{4}+16{\left(\Omega^{2}-\omega_{j}^{2}\right)}^{2}+8\beta^{2}(\Omega^{2}+\omega_{j}^{2})}.$$

**Figure 2:**
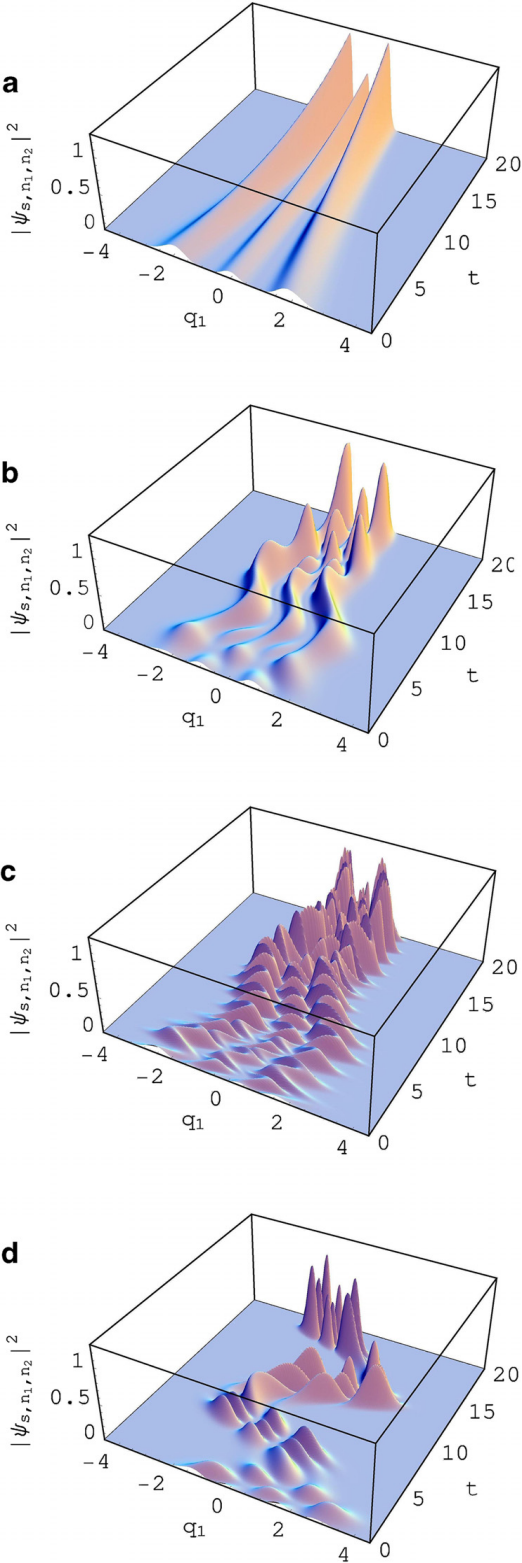
**Probability density (A).** This represents the probability density $|\psi_{s,n_{1},n_{2}}(q_{1},q_{2},t){|}^{2}$ as a function of *q*_1_ and *t*. Here, we did not take into account the squeezing effect (i.e., we let *r*_1_=*r*_2_=0). Various values we have taken are *q*_2_=0, *n*_1_=*n*_2_=2, ℏ=1, *R*_0_=*R*_1_=*R*_2_=0.1, *L*_0_=*L*_1_=*L*_2_=1, *C*_1_=1, *C*_2_=1.2, *p*_1*c*_(0) = *p*_2*c*_(0) = 0, and *δ* = 0. The values of $\left(q_{1c}(0),q_{2c}(0),E_{0},\Omega \right)$ are (0,0,0,0) **(a)**, (0.5,0.5,0,0) **(b)**, (0.5,0.5,10,4) **(c)**, and (0.5,0.5,0.5,0.53) **(d)**. All values are taken dimensionlessly for convenience: this convention will be used in all subsequent figures.

**Figure 3:**
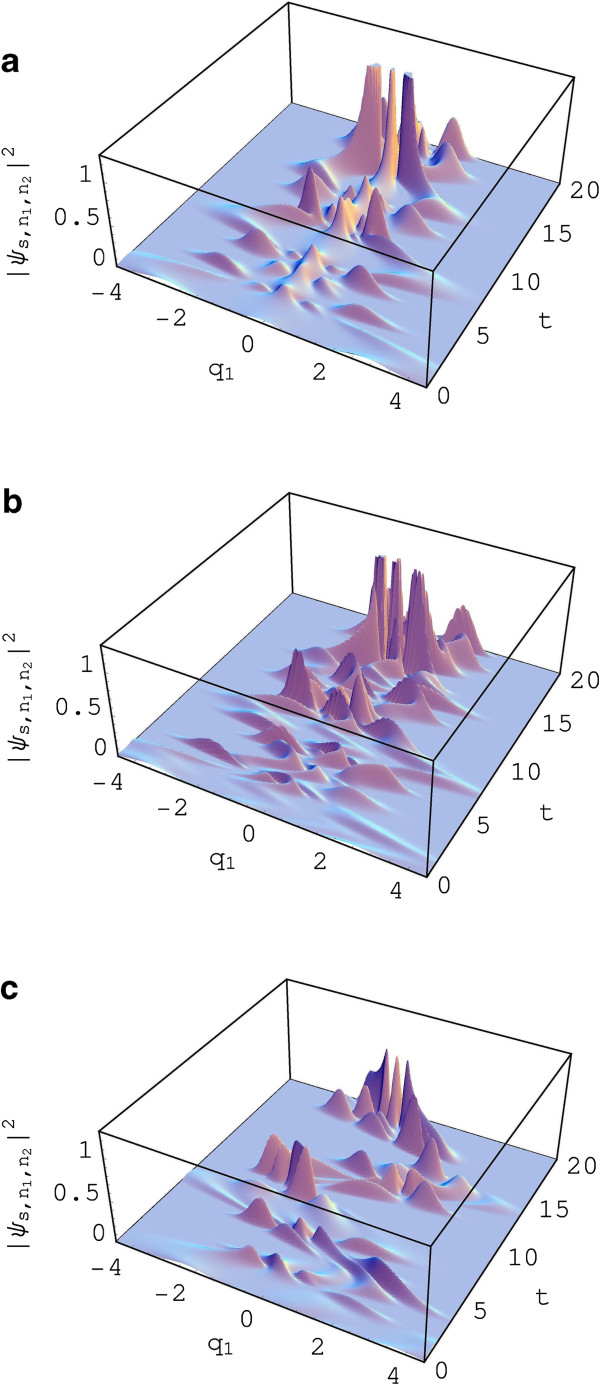
**Probability density (B).** The probability density $|\psi_{s,n_{1},n_{2}}(q_{1},q_{2},t){|}^{2}$ with squeezing parameters *r*_1_ = *r*_2_ = 0.7 and *ϕ*_1_ = *ϕ*_2_ = 1.5 is shown here as a function of *q*_1_ and *t*. Various values we have taken are *q*_2_ = 0, *n*_1_ = *n*_2_ = 2, ℏ=1, *R*_0_ = *R*_1_ = *R*_2_ = 0.1, *L*_0_ = *L*_1_ = *L*_2_ = 1, *C*_1_ = 1, *C*_2_ = 1.2, *p*_1*c*_(0) = *p*_2*c*_(0) = 0, and *δ* = 0. The values of $\left(q_{1c}(0),q_{2c}(0),E_{0},\Omega \right)$ are (0,0,0,0) **(a)**, (0.5,0.5,10,4) **(b)**, and (0.5,0.5,0.5,0.53)**(c)**.

The probability densities $|\psi_{s,n_{1},n_{2}}(q_{1},q_{2},t){|}^{2}$ are plotted in Figures 2 and 3 as a function of *q*_1_ and *t* under this circumstance. As time goes by, the overall probability densities gradually converge to the origin where *q*_1_=0 due to the dissipation of energy caused by the existence of resistances in the circuit. If there are no resistances in the circuit, the probability densities no longer converge with time. An electronic system in general loses energy by the resistances, and the lost energy changes to thermal energy. Actually, Figure 2 belongs to DN due to the condition *r*_1_=*r*_2_=0 supposed in it. The wave function used in Figure 2a is not displaced and is consequently the same as that of the number state. Figure 2b is distorted by the effect of displacement. From Figure 2c,d, you can see that the exertion of a sinusoidal power source gives additional distortion. The frequency of E(t) is relatively large for Figure 2c whereas it is small for Figure 2d.

You can see the effects of squeezing from Figure 3. The probability densities in the DSN are more significantly distorted than those of the DN. We can see from Figure 3b,c that the time behavior of probability densities is highly affected by external power source. If there is no power source in the circuit, the displacement of charge, specified with an initial condition, may gradually disappear according to its dissipation induced by resistances in the circuit. This is the same as that interpreted from the DN and exactly coincides with classical analysis of the system.

While various means and technologies to generate squeezed and/or displaced light are developed in the context of quantum optics after the seminal work of Slusher et al. [31] for observing squeezed light in the mid 1980s, (displaced) squeezed number state with sufficient degree of squeezing for charges and currents in a circuit quantum electrodynamics is first realized not long ago by Marthaler et al. [32] as far as we know. The circuit they designed not only undergoes sufficiently low dissipation but its potential energy also contains a positive quartic term that leads to achieving strong squeezing. Another method to squeeze quantum states of mechanical oscillation of charge carriers in a circuit is to use the technique of back-action evasion [33,34] that is originally devised in order to measure one of two arbitrary conjugate quadratures with high precision beyond the standard quantum limit.

Though it is out of the scope of this work, the superpositions of any two DSNs may also be interesting topics to study, thanks to their nonclassical features that have no classical analogues. The quantum properties such as quadrature squeezing, quantum number distribution, purity, and the Mandel *Q* parameter for the superposition of two DSNs out of phase with respect to each other are studied in the literatures (see, for example, [35]).

### Quantum fluctuations

Now let us see the quantum fluctuations and uncertainty relations for charges and currents in the DSN for the original system. It is well known that quantum energy and any physical observables are temporarily changed due to their quantum fluctuations. The theoretical study for the origin and background physics of quantum fluctuations have been performed in [36] by introducing stochastic and microcanonical quantizations.

If we consider the method of consecutive unitary transformation, the expectation value for an arbitrary operator ${\hat{O}}_{j}$ in the original system can be evaluated from

(57) $$\;\begin{array}{l} \;\langle \psi_{s,n_{1},n_{2}}(t)|{\hat{O}}_{j}|\psi_{s,n_{1},n_{2}}(t)\rangle \\ =\;\langle \psi_{n_{1},n_{2}}^{B}(0)|{Ŝ}_{j}^{‡}{\hat{D}}_{j}^{‡}{\hat{T}}_{\text{Bj}}^{‡}{Û}_{B}^{‡}{Û}_{A}^{‡}{\hat{O}}_{j}{Û}_{A}{Û}_{B}{\hat{T}}_{\text{Bj}}{\hat{D}}_{j}{Ŝ}_{j}|\psi_{n_{1},n_{2}}^{B}(0)\rangle . \end{array}$$

Using this relation, the expectation value of charges ${\hat{q}}_{j}$ and currents ${\hat{p}}_{j}$ is derived to be

(58) $$\;\begin{array}{l} \langle \psi_{s,n_{1},n_{2}}(t)|{\hat{q}}_{1}|\psi_{s,n_{1},n_{2}}(t)\rangle \\ \;=\sqrt[{4}]{\frac{C_{1}}{C_{2}}}e^{-\mathrm{\beta t}/2}[Y_{1}(t)\cos\varphi \;+\;Y_{2}(t)\sin\varphi ], \end{array}$$

(59) $$\;\begin{array}{l} \langle \psi_{s,n_{1},n_{2}}(t)|{\hat{q}}_{2}|\psi_{s,n_{1},n_{2}}(t)\rangle \\ \;=\sqrt[{4}]{\frac{C_{2}}{C_{1}}}e^{-\mathrm{\beta t}/2}[\;-Y_{1}(t)\sin\varphi \;+\;Y_{2}(t)\cos\varphi ], \end{array}$$

(60) $$\;\begin{array}{l} \langle \psi_{s,n_{1},n_{2}}(t)|{\hat{p}}_{1}|\psi_{s,n_{1},n_{2}}(t)\rangle \\ \;=-\sqrt[{4}]{\frac{C_{2}}{C_{1}}}e^{\mathrm{\beta t}/2}[Y_{1}(t)\cos\varphi +Y_{2}(t)\sin\varphi ], \end{array}$$

(61) $$\;\begin{array}{l} \langle \psi_{s,n_{1},n_{2}}(t)|{\hat{p}}_{2}|\psi_{s,n_{1},n_{2}}(t)\rangle \\ \;=\sqrt[{4}]{\frac{C_{1}}{C_{2}}}e^{\mathrm{\beta t}/2}[Y_{1}(t)\sin\varphi -Y_{2}(t)\cos\varphi ], \end{array}$$

where

(62) $$\;Y_{j}(t)=\sqrt{\frac{\hbar }{2\mu_{j}\omega_{j}}}(\alpha_{j}e^{-i\omega_{j}t}+\alpha_{j}^{*}e^{i\omega_{j}t})+q_{\text{jp}}(t),$$

(63) $$\;\begin{array}{l} Y_{j}(t)\;=\;\sqrt{\frac{\mu_{j}\hbar }{2\omega_{j}}}\left[\alpha_{j}e^{-i\omega_{j}t}(\beta /2\;+\;i\omega_{j})\;+\;\alpha_{j}^{*}e^{i\omega_{j}t}(\beta /2\;-\;i\omega_{j})\right]\;-p_{\text{jp}}(t). \end{array}$$

The expectation value of square of ${\hat{q}}_{j}$ and ${\hat{p}}_{j}$ can also be obtained form the same method, and we listed them in Appendix Appendix 3. In fact, Equations 58 and 59 are the same as the classically predicted amount of charges *q*_cl,1_ and *q*_cl,2_ in *C*_1_ and *C*_2_ in the original system, respectively. If we consider that *α*_*j*_ are given by Equation 36, *q*_cl,1_ and *q*_cl,2_ can be rewritten, after a little evaluation, in the form

(64) $$\begin{array}{ll} q_{\text{cl},1}=\sqrt[{4}]{\frac{C_{1}}{C_{2}}}e^{-\mathrm{\beta t}/2} & \{[q_{1c}(t)+q_{1p}(t)]\cos\varphi \\ & +[q_{2c}(t)+q_{2p}(t)]\sin\varphi \}, \end{array}$$

(65) $$\begin{array}{ll} q_{\text{cl},2}=\sqrt[{4}]{\frac{C_{2}}{C_{1}}}e^{-\mathrm{\beta t}/2} & \{[q_{2c}(t)+q_{2p}(t)]\cos\varphi \\ & -[q_{1c}(t)+q_{1p}(t)]\sin\varphi \}. \end{array}$$

**Figure 4:**
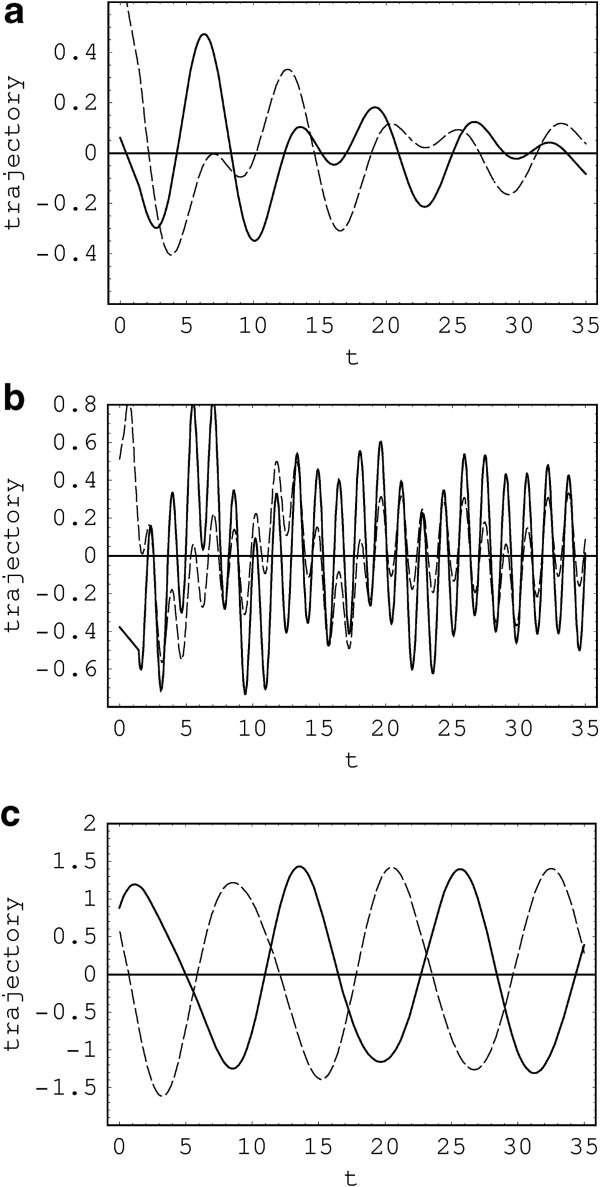
**Classically predicted amount of charges in capacitors.** This illustration represents the time behavior of *q*_cl,1_ (thick solid line) and *q*_cl,2_ (dashed line) where *R*_0_ = *R*_1_ = *R*_2_ = 0.1, *L*_0_ = *L*_1_ = *L*_2_ = 1, *C*_1_ = 1, *C*_2_ = 1.2, *q*_1*c*_(0) = *q*_2*c*_(0) = 0.5, *p*_1*c*_(0) = *p*_2*c*_(0) = 0, and *δ* = 0. The values of $\left(E_{0},\Omega \right)$ are (0,0) **(a)**, (10,4) **(b)**, and (0.5,0.53) **(c)**.

We illustrated *q*_cl,1_ and *q*_cl,2_ in Figure 4 as a function of time. To understand the time behavior of these quantities, it may be worth to recall that complementary functions, *q*_*jc*_(*t*), and particular solutions, *q*_*jp*_(*t*), are not associated to the original system but to the firstly transformed system. We can also easily confirm from similar evaluation that the time behavior of canonical conjugate currents *p*_cl,*j*_ are represented in terms of *q*_*jc*_(*t*), *p*_*jc*_(*t*), and *p*_*jp*_(*t*) (see Appendix Appendix 4).

The definition of quantum fluctuations for any quantum operator ${\hat{O}}_{j}$ in the DSN is given by

(66) $$\begin{array}{ll} {\left(\Delta {\hat{O}}_{j}\right)}_{s}\;\;=\;\; & \left[\langle \psi_{s,n_{1},n_{2}}(t)|{\hat{O}}_{j}^{2}|\psi_{s,n_{1},n_{2}}(t)\rangle \right)\\ & {\left(-{\left(\langle \psi_{s,n_{1},n_{2}}(t)|{\hat{O}}_{j}|\psi_{s,n_{1},n_{2}}(t)\rangle \right)}^{2}\right]}^{1/2}. \end{array}$$

Using this, we obtain the fluctuations of charges and currents as

(67) $$\;\begin{array}{ll} {\left(\Delta {\hat{q}}_{1}\right)}_{s}=\sqrt{\frac{\hbar }{2}}\sqrt[{4}]{\frac{C_{1}}{C_{2}}}e^{-\mathrm{\beta t}/2} & \left[(2n_{1}\;+\;1)\frac{F_{1}(t)}{\mu_{1}\omega_{1}}\overset{2}{\cos}\varphi \right)\\ & \;+{\left((2n_{2}\;+\;1)\frac{F_{2}(t)}{\mu_{2}\omega_{2}}\overset{2}{\sin}\varphi \;\right]}^{1/2}, \end{array}$$

(68) $$\begin{array}{ll} {\left(\Delta {\hat{q}}_{2}\right)}_{s}\;=\;\sqrt{\frac{\hbar }{2}}\sqrt[{4}]{\frac{C_{2}}{C_{1}}}e^{-\mathrm{\beta t}/2} & \left[(2n_{1}\;+\;1)\frac{F_{1}(t)}{\mu_{1}\omega_{1}}\overset{2}{\sin}\varphi \right)\\ & \;+{\left((2n_{2}+1)\frac{F_{2}(t)}{\mu_{2}\omega_{2}}\overset{2}{\cos}\varphi \;\right]}^{1/2}\;,\;\; \end{array}$$

(69) $$\;\begin{array}{ll} {\left(\Delta {\hat{p}}_{1}\right)}_{s}\;=\;\sqrt{\frac{\hbar }{2}}\sqrt[{4}]{\frac{C_{2}}{C_{1}}}e^{\mathrm{\beta t}/2} & \left[(2n_{1}\;+\;1)\frac{\mu_{1}F_{1}(t)}{\omega_{1}}\overset{2}{\cos}\varphi \right)\\ & \;+{\left((2n_{2}\;+1)\frac{\mu_{2}F_{2}(t)}{\omega_{2}}\overset{2}{\sin}\varphi \;\right]}^{1/2}\;,\;\; \end{array}$$

(70) $$\;\begin{array}{ll} {\left(\Delta {\hat{p}}_{2}\right)}_{s}\;=\;\sqrt{\frac{\hbar }{2}}\sqrt[{4}]{\frac{C_{1}}{C_{2}}}e^{\mathrm{\beta t}/2} & \left[(2n_{1}\;+\;1)\frac{\mu_{1}F_{1}(t)}{\omega_{1}}\overset{2}{\sin}\varphi \right)\\ & \;+{\left((2n_{2}\;+\;1)\frac{\mu_{2}F_{2}(t)}{\omega_{2}}\overset{2}{\cos}\varphi \;\;\right]}^{1/2}\;.\;\; \end{array}$$

As we have seen before, the expectation values associated to charges and currents are represented in terms of complementary functions, *q*_*jc*_(*t*) and *p*_*jc*_(*t*), and particular solutions *q*_*jp*_(*t*) and *p*_*jp*_(*t*). The amplitude of complementary functions is determined from the strength of displacements, whereas the particular solutions are determined by the power source E(t) (see Equations 19 and 20). However, all of the fluctuations do not involve such solutions. This means that the displacement and the electric power source do not affect to the fluctuations of charges and currents.

The uncertainty products ${\left(\Delta {\hat{q}}_{j}\right)}_{s}{\left(\Delta {\hat{p}}_{j}\right)}_{s}$ between charges and their conjugate currents can be easily identified by means of Equations 67 to 70. For the case of the DN that are given from the limit *r*_1_=*r*_2_→0, we have *F*_1_=*F*_2_=0 and $F_{j}=\omega_{j}^{2}+\beta^{2}/4$. Then, the uncertainty products become

(71) $$\begin{array}{ll} {\left(\Delta {\hat{q}}_{1}\right)}_{c}{\left(\Delta {\hat{p}}_{1}\right)}_{c} & =\frac{\hbar }{2}\left\{\left(1+\frac{\beta^{2}}{4\omega_{1}^{2}}\right){\left(2n_{1}+1\right)}^{2}\overset{4}{\cos}\varphi \right)\\ & \;+\left(1+\frac{\beta^{2}}{4\omega_{2}^{2}}\right){\left(2n_{2}+1\right)}^{2}\overset{4}{\sin}\varphi \\ & \;+\left[\frac{\mu_{1}\omega_{1}}{\mu_{2}\omega_{2}}+\frac{\mu_{2}\omega_{2}}{\mu_{1}\omega_{1}}+\frac{\beta^{2}}{4\omega_{1}\omega_{2}}\left(\frac{\mu_{2}}{\mu_{1}}+\frac{\mu_{1}}{\mu_{2}}\right)\right]\\ & \;\times {\left(\frac{\left(2n_{1}+1)(2n_{2}+1\right)}{4}\overset{2}{\sin}(2\varphi )\right\}}^{1/2}, \end{array}$$

(72) $$\begin{array}{ll} {\left(\Delta {\hat{q}}_{2}\right)}_{c}{\left(\Delta {\hat{p}}_{2}\right)}_{c} & =\frac{\hbar }{2}\left\{\left(1+\frac{\beta^{2}}{4\omega_{1}^{2}}\right){\left(2n_{1}+1\right)}^{2}\overset{4}{\sin}\varphi \right)\\ & \;+\left(1+\frac{\beta^{2}}{4\omega_{2}^{2}}\right){\left(2n_{2}+1\right)}^{2}\overset{4}{\cos}\varphi \\ & \;+\left[\frac{\mu_{1}\omega_{1}}{\mu_{2}\omega_{2}}+\frac{\mu_{2}\omega_{2}}{\mu_{1}\omega_{1}}+\frac{\beta^{2}}{4\omega_{1}\omega_{2}}\left(\frac{\mu_{2}}{\mu_{1}}+\frac{\mu_{1}}{\mu_{2}}\right)\right]\\ & \;\times {\left(\frac{\left(2n_{1}+1)(2n_{2}+1\right)}{4}\overset{2}{\sin}(2\varphi )\right\}}^{1/2}. \end{array}$$

**Figure 5:**
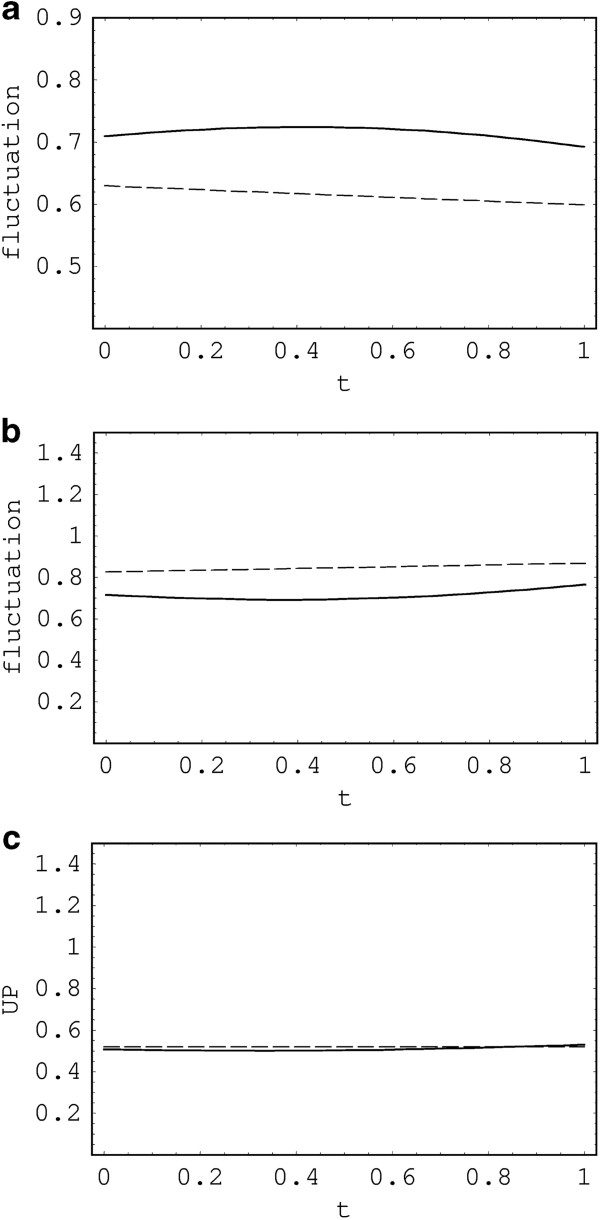
**Fluctuations.** This inset shows fluctuations ${\left(\Delta {\hat{q}}_{1}\right)}_{c}$ (dashed line) and ${\left(\Delta {\hat{q}}_{1}\right)}_{s}$ (thick solid line) **(a)**, and ${\left(\Delta {\hat{p}}_{1}\right)}_{c}$ (dashed line) and ${\left(\Delta {\hat{p}}_{1}\right)}_{s}$ (thick solid line) **(b)**, and uncertainty product ${\left(\Delta {\hat{q}}_{1}\right)}_{c}{\left(\Delta {\hat{p}}_{1}\right)}_{c}$ (dashed line) and ${\left(\Delta {\hat{q}}_{1}\right)}_{s}{\left(\Delta {\hat{p}}_{1}\right)}_{s}$ (thick solid line) **(c)** as a function of *t* where *n*_1_=*n*_2_=0, ℏ=1, *R*_0_ = *R*_1_ = *R*_2_ = 0.1, *L*_0_ = *L*_1_ = *L*_2_ = 1, *C*_1_ = 1, and *C*_2_ = 1.2. The values of squeezing parameters for the DSN are *r*_1_ = 0.1, *r*_2_ = 0.3, *ϕ*_1_ = 1.2, and *ϕ*_2_ = 0.6.

These are the same as the uncertainty products in the number states and are always larger than ℏ/2, preserving the uncertainty principle. Thus, we can conclude that the uncertainty products in the DN are the same as those of the ordinary number states. Evidently, the uncertainty principle is inherent in quantum mechanical context described by canonical variables. The results, Equations 71 and 72 with *n*_1_=*n*_2_=0, are exactly the same as Equations 29 and 30 of [4], respectively. Moreover, for *R*_1_=*R*_2_=*R*_3_→0 (i.e., *β*→0), the above two equations reduce to Equations 52 and 53 in [6], which are evaluated in ordinary number state. Hence, this work includes all the results of both [4] (no power source) and [6] (no resistances) as special cases. The fluctuations and uncertainty product in the DN and in the DSN are plotted in Figure 5. We can adjust the uncertainty (or fluctuation) of a quadrature to be small at the expense of broadening that of another quadrature, or vice versa. The uncertainty ${\left(\Delta {\hat{q}}_{1}\right)}_{s}$ in the case of this figure is larger than ${\left(\Delta {\hat{q}}_{1}\right)}_{c}$, while ${\left(\Delta {\hat{p}}_{1}\right)}_{s}$ is smaller than ${\left(\Delta {\hat{p}}_{1}\right)}_{c}$ due to the squeezing effect. Therefore, it is relatively difficult for us to know the precise value of charge *q*_1_, while we can find out the conjugate current *p*_1_ more precisely. However, the relevant uncertainty product in the DSN is nearly unaltered from that in the DN.

## Conclusions

In summary, the time evolution of the DSN for the two-dimensional electronic circuit composed of nanoscale elements and driven by a power source is investigated using unitary transformation method. Two steps of the unitary transformation are executed: We removed the cross term involving ${\hat{p}}_{1}{\hat{p}}_{2}$ in the original Hamiltonian from the first step, and the linear terms represented in terms of ${\hat{q}}_{j}E(t)$ in the firstly transformed Hamiltonian are eliminated by second unitary transformation.

We can see from Equation 6 that the original Hamiltonian is time-dependent. When treating a time-dependent Hamiltonian system dynamically, one usually employs classical solutions of the equation of motion for a given system (or for a system similar to a given system) [6,7]. We also introduced such classical solutions in Equations 19 to 20 and in Equations 47 to 48. Among them, particular solutions *q*_*jp*_ and *p*_*jp*_ are important in developing quantum theory of the system involving external power source since they are crucial factors that lead the transformed Hamiltonian to be simple so that we can easily treat it.

Since the transformed system is just the same as the one that consists of two independent simple harmonic oscillators, provided that we can neglect the trivial terms $L_{\text{jp}}(t)$ in the transformed Hamiltonian, we easily identified the complete quantum solutions in the DSN in the transformed system. We also obtained the wave functions of the DSN in the original system via the technique of inverse transformation, as shown in Equation 50. If we regard the fact that the probability does not reflect the phase of a wave function, the overall phase of these states is relatively unimportant for many cases. However, in some applications such as the computation of expectation values using generating or characteristic functions given in [17], the exact knowledge of overall phase is crucial. For *r*_1_=*r*_2_=0, the wave function in the DSN exactly reduces to that of the DN.

We analyzed the probability densities in the DN and in the DSN from Figures 2 and 3, respectively, with the choice of sinusoidal signal source. The probability densities in the DN given in Figure 2b,c,d oscillate with time. Moreover, their time behaviors are more or less distorted. The probability density, however, does not oscillate when there are no displacement and no signal of power source (see Figure 2a). The probability densities in the DSN are distorted much more significantly than those of the DN.

The time behavior of probability densities of quantum states, both the DN and the DSN, is highly affected by external driving power source. When there is no external power source(E(t)=0), the displacement of charges, specified with a certain initial condition, gradually disappears as time goes by like a classical state.

The fluctuations and uncertainty products of charges and currents are derived in the DSN, and it is shown that their value is independent of the size of the particular solutions *q*_*jp*_(*t*) and *p*_*jp*_(*t*). From this, together with the fact that *q*_*jp*_(*t*) and *p*_*jp*_(*t*) are determined by the characteristics of E(t), it is clear that the electric power source does not affect on the fluctuation of canonical variables. If we ignore the time dependence of *F*_*j*_(*t*) and $F_{j}(t)$, ${\left(\Delta {\hat{q}}_{j}\right)}_{s}$ decrease exponentially with time, whereas ${\left(\Delta {\hat{p}}_{j}\right)}_{s}$ increase exponentially.

From Equations 64 and 65, we can see that the time behavior of *q*_*j*_ is determined by two factors: One is displacement and the other is the signal of power source. For better understanding of this, recall that the amplitude of complementary functions gives displacement of the system, and the particular solutions are closely related to external driving force (i.e., in this case, the power source).

In this paper, we did not consider thermal effects for the system. The thermal effects, as well as dissipation, may be worth to be considered in the studies of quantum fluctuations of electronic circuits with nanosize elements because the practical circuits are always working in thermal states with the presence of damping. It may therefore be a good theme to investigate DSNs with thermalization as a next task, and we plan to investigate it in the near future.

## Appendix 1

### The eighth formula of 7.374 in [29]

(73) $$\begin{array}{l} \int_{-\infty }^{\infty }e^{-{\left(x-y\right)}^{2}}H_{n}(\text{ax})\text{dx}=\Pi^{1/2}{\left(1-a^{2}\right)}^{n/2}H_{n}\left(\frac{\text{ay}}{{\left(1-a^{2}\right)}^{1/2}}\right). \end{array}$$

## Appendix 2

### Correction of Equation 45 of [30]

The second line of Equation 45 of [30] needs to be corrected as

(74) $$\begin{array}{l} \;\exp\left(\cdots +x\frac{(x_{0}F_{2}+ip_{0})\cos t}{B}-\cdots \;\right)\\ \to \exp\left(\cdots +x\frac{x_{0}F_{2}+ip_{0}}{B}-\cdots \;\right). \end{array}$$

Besides, among various functions that appeared in Equation 45 of [30], $F_{3}$ (Equation 23) and *A* (Equation 46) should be altered as

(75) $$\begin{array}{ll} & F_{3}=\frac{\cosh r+e^{-\mathrm{i\phi}}\sin\phi \sinh r}{\cosh r+e^{\mathrm{i\phi}}\sin\phi \sinh r}\\ \to & F_{3}=\frac{\cosh r+e^{-\mathrm{i\phi}}\sinh r}{\cosh r+e^{\mathrm{i\phi}}\sinh r}, \end{array}$$

(76) $$\begin{array}{ll} & A=\left(1-\frac{2i\sin t}{F_{4}^{2}B}\right)\frac{B-2i\sin t/\overset{2}{\underset{4}{F}}}{B}\\ \to & A=1-\frac{2i\sin t}{F_{4}^{2}B}. \end{array}$$

For the convenience of comparison, we provide a list of correspondences between our notations and the notations used in [30]:

(77) $$\begin{array}{ll} \begin{array}{l} G_{\text{aj}}\Leftrightarrow F_{1},\;\;\;G_{\text{bj}}\Leftrightarrow F_{3},\;\;\;G_{\text{cj}}\Leftrightarrow {\left(F_{4}\right)}^{2},\\ G_{\text{dj}}\Leftrightarrow F_{2},\;\;\;h_{\text{aj}}\Leftrightarrow B,\;\;\;h_{\text{bj}}\Leftrightarrow \mathrm{A.} \end{array} & \; \end{array}$$

## Appendix 3

### Expectation value of ${\hat{q}}_{j}^{2}$ and ${\hat{p}}_{j}^{2}$

According to the rule, Equation 57, for evaluating expectation values, we also have the expectation value of square of charges and currents as

(78) $$\begin{array}{cc} \langle \psi_{s,n_{1},n_{2}}(t)|{\hat{q}}_{1}^{2}|\psi_{s,n_{1},n_{2}}(t)\rangle =\sqrt{\frac{C_{1}}{C_{2}}}e^{-\mathrm{\beta t}}\left[\left(\frac{\hbar }{2\mu_{1}\omega_{1}}(2n_{1}+1)F_{1}(t)+Y_{1}^{2}(t)\right)\overset{2}{\cos}\varphi \right)\\ \left(+\left(\frac{\hbar }{2\mu_{2}\omega_{2}}(2n_{2}+1)F_{2}(t)+Y_{2}^{2}(t)\right)\overset{2}{\sin}\varphi +Y_{1}(t)Y_{2}(t)\sin(2\varphi )\right], & \end{array}$$

(79) $$\begin{array}{cc} \langle \psi_{s,n_{1},n_{2}}(t)|{\hat{q}}_{2}^{2}|\psi_{s,n_{1},n_{2}}(t)\rangle =\sqrt{\frac{C_{2}}{C_{1}}}e^{-\mathrm{\beta t}}\left[\left(\frac{\hbar }{2\mu_{1}\omega_{1}}(2n_{1}+1)F_{1}(t)+Y_{1}^{2}(t)\right)\overset{2}{\sin}\varphi \right)\\ +\left(\left(\frac{\hbar }{2\mu_{2}\omega_{2}}(2n_{2}+1)F_{2}(t)+Y_{2}^{2}(t)\right)\overset{2}{\cos}\varphi -Y_{1}(t)Y_{2}(t)\sin(2\varphi )\right], & \end{array}$$

(80) $$\begin{array}{cc} \langle \psi_{s,n_{1},n_{2}}(t)|{\hat{p}}_{1}^{2}|\psi_{s,n_{1},n_{2}}(t)\rangle =\sqrt{\frac{C_{2}}{C_{1}}}e^{\mathrm{\beta t}}\left[\left(\frac{\mu_{1}\hbar }{2\omega_{1}}(2n_{1}+1)F_{1}(t)+Y_{1}^{2}(t)\right)\overset{2}{\cos}\varphi \right)\\ +\left(\left(\frac{\mu_{2}\hbar }{2\omega_{2}}(2n_{2}+1)F_{2}(t)+Y_{2}^{2}(t)\right)\overset{2}{\sin}\varphi +Y_{1}(t)Y_{2}(t)\sin(2\varphi )\right], & \end{array}$$

(81) $$\begin{array}{cc} \langle \psi_{s,n_{1},n_{2}}(t)|{\hat{p}}_{2}^{2}|\psi_{s,n_{1},n_{2}}(t)\rangle =\sqrt{\frac{C_{1}}{C_{2}}}e^{\mathrm{\beta t}}\left[\left(\frac{\mu_{1}\hbar }{2\omega_{1}}(2n_{1}+1)F_{1}(t)+Y_{1}^{2}(t)\right)\overset{2}{\sin}\varphi \right)\\ +\left(\left(\frac{\mu_{2}\hbar }{2\omega_{2}}(2n_{2}+1)F_{2}(t)+Y_{2}^{2}(t)\right)\overset{2}{\cos}\varphi -Y_{1}(t)Y_{2}(t)\sin(2\varphi )\right], & \end{array}$$

where

(82) $$F_{j}(t)=\cosh(2r_{j})+\sinh(2r_{j})\cos(\phi_{j}-2\omega_{j}t),$$

(83) $$\begin{array}{ll} F_{j}(t) & =(\beta^{2}/4+\omega_{j}^{2})\cosh(2r_{j})+[(\beta^{2}/4-\omega_{j}^{2})\cos(\phi_{j}-2\omega_{j}t)\\ & \;-\beta \omega_{j}\sin(\phi_{j}-2\omega_{j}t)]\sinh(2r_{j}). \end{array}$$

## Appendix 4

### Classical currents

Through the same vein as that of the calculation of *q*_cl,1_ and *q*_cl,2_ given in Equations 64 and 65, we can evaluate classical currents *p*_cl,1_ and *p*_cl,2_ from their quantum expectation value given in Equations 60 and 61. Thus, we have

(84) $$\begin{array}{rcll} p_{\text{cl},1} & = & \sqrt[{4}]{\frac{C_{2}}{C_{1}}}e^{\mathrm{\beta t}/2}\{[p_{1c}(t)-\mu_{1}\beta q_{1c}(t)/2+p_{1p}(t)]\cos\varphi & \\ & & +[p_{2c}(t)-\mu_{2}\beta q_{2c}(t)/2+p_{2p}(t)]\sin\varphi \}, & \end{array}$$

(85) $$\begin{array}{rcll} p_{\text{cl},2} & = & \sqrt[{4}]{\frac{C_{1}}{C_{2}}}e^{\mathrm{\beta t}/2}\{[p_{2c}(t)-\mu_{2}\beta q_{2c}(t)/2+p_{2p}(t)]\cos\varphi & \\ & & -[p_{1c}(t)-\mu_{1}\beta q_{1c}(t)/2+p_{1p}(t)]\sin\varphi \}. & \end{array}$$

## Abbreviations

DN: displaced number state.

## Competing interests

The authors declare that they have no competing interests.
